# Operative treatment of acute acromioclavicular joint injuries graded Rockwood III and IV: risks and benefits in tight rope technique vs. k-wire fixation

**DOI:** 10.1186/1754-9493-7-18

**Published:** 2013-05-30

**Authors:** Klemens Horst, Thomas Dienstknecht, Miguel Pishnamaz, Richard Martin Sellei, Philipp Kobbe, Hans-Christoph Pape

**Affiliations:** 1 Department of Orthopedic Trauma Surgery, University Hospital RWTH Aachen, Paulwelsstreet 30, Aachen, 52074, Germany

**Keywords:** Tight rope technique, K-wires, Costs, Acromioclavicular joint dislocation, Reconstruction, Surgery, Comparison, Analysis

## Abstract

**Background:**

Operative treatment of acromioclavicular joint injuries is recommended for higher degree dislocations. Recently a new option has become available with the minimally-invasive tight rope technique. Whereas clinical studies justify the medical use, risks and benefits remain unclear. Therefore, this study analyzed these facts associated with this procedure and compared them to K-wire fixation.

**Material and Methods:**

A retrospective analysis was performed of patients surgically treated either with the TightRope™-technique (TR) or K-wires (KW) for a first event isolated Rockwood type III or higher acromioclavicular joint dislocation between 2004 and 2011. Timing for surgery, surgical duration, length of hospital stay, costs, complications and outpatient visits were recorded.

**Results:**

41 patients were included (TR: n = 18; KW: n = 23) with comparable demographics and injury severity. A trend towards shorter operation time was seen in the TR group (TR: 64.3 ±19.8 min. vs. KW: 80.9 ±33.7 min., n.s.) A tendency for lower total operation theater costs was seen in the TR group (TR: 474 ±436.5€ vs. KW: 749.1 ±31.2€, n.s.). Patients from the TR group left hospital earlier (TR: 2 ±1d vs. KW: 3.6 ±1.8d, p = 0.002). Severe complications (i.e. a fracture of the clavicle or nerve damage) occurred in neither of the groups. Early loss of reduction (n = 1) and impaired wound healing (n = 2) was seen in the TR group. Migrating K-wires (n = 4), loss of reduction (n = 1) and impingement syndrome (n = 1) were recorded in the KW group.

**Conclusion:**

Usage of the tight rope technique offered advantages, such as being a safe minimally-invasive technique and showed a tendency towards shorter operation time, and lower physician- and total operation and theater costs. Material costs were significantly higher for this device but patients were discharged earlier. The influence of different clinical long-term results on the financial outcome needs to be evaluated in further studies.

## Introduction

The acromioclavicular joint luxation (ACJ-luxation) typically affects young adults [1]. The cause is usually found to be as a result of a direct blow to the shoulder. The indirect pathomechanism with an extended arm is rare [2]. Treatment of ACJ-luxation is guided by the Rockwood classification [3], and operative treatment is typically performed in higher grade ACJ-luxations [4]. Various methods are described, such as an augmented sutures with absorbable materials, stabilization via K-wires in combination with or without additional wire loops [5], hook plates [6] or the Bosworth screw [7,8].

A relatively new option is offered by the TightRope™ system (Arthrex, Naples, USA). This technique was developed as a minimally-invasive procedure to treat the torn conoid and trapezoid ligaments in acromioclavicular luxation [9]. Due to its minimally-invasive approach, traumatic soft tissue damage is reduced in comparison with open surgery procedures. Also, advantages such as the lack of a need for re-operation for the removal of hardware, such as wires, screws or plates, are striking. Complications of hardware failure, like breakage, dislocation or bone resorption, causing re-operation and further harming of the patient should be minimized [10,11]. Also better cosmetic results are reported [12]. A modified arthroscopic technique furthermore supports the investigation of concomitant injuries of the glenohumeral joint.

There is a considerable number of publications investigating the operative treatment of established methods [13-15]. Results comparing different methods are often focused on biomechanical results [16,17]. Publications regarding treatment by the tight rope technique are increasing, but published studies recording operative and clinical outcomes are rare [12,18,19]. While these first clinical results are encouraging and the use of the tight rope technique is increasing, it remains unclear whether or not this new device is cost-effective and what complications do occur.

We hypothesize that due a sophisticated surgical procedure, non-reusable devices and uncertainty in follow up treatment in patients with the tight rope technique, costs and surgical duration rise as well as hospital stay will extend and follow up visits will be more frequent.

The purpose of the present study was to determine whether this new device is beneficial from an economic and patient safety point of view or not. Therefore, patients from our institution that were treated by the tight rope technique (TR) were analyzed and the results were compared to patients that were treated with K-wire fixation (KW). The focus was on the timing of surgical treatment, length and costs of hospital stay, diagnostic costs, operation time and operation costs (anesthesia, physicians payment, material costs), and the proceeds for stationary and outpatient treatment as well as complications that occur.

## Material and methods

### Patient population

A retrospective study was performed that included consecutive patients who were diagnosed and treated at our institution between January 1^st^ in 2004 and December 31^st^ in 2011. The inclusion criteria were a first injury event, with a grading of Rockwood type III or higher, whether the injury occurred less than three weeks previously, an absence of concomitant shoulder injury or previous surgery for acromioclavicular joint luxation and full accounting data.

Furthermore, only patients that received K-wire fixation or that were treated by the TightRope™ system were included. A decision regarding operative procedure was made by the patient and surgeon after the patient was informed about the risks and benefits of both surgical techniques. All patients gave their written informed consent before undergoing the operation. All demographic data, clinical and operative information as well as time intervals were taken from a retrospective patients chart review.

### Surgical techniques

All patients were operated on under general anesthesia and in the beach chair position. Intraoperative fluoroscopy was used and patients received single shot antibiotics before operation started. Using the TightRope™ system (Arthrex, Naples/USA), a minimally-invasive technique, a non-absorbable string was positioned through boreholes between the coracoid process and the clavicle. A reduction was performed and the string was held in position by anchors placed underneath the coracoid process and above the clavicle [2]. Another established method is stabilization via K-wires. K-wires (strength 1.6 mm) were brought in from the lateral aspect of the acromion aiming cranially on the center of the lateral clavicle. The clavicle was then brought into position and the K-wires were drilled in, followed by the reconstruction of the torn ligaments. The wires were removed about six weeks later.

### Postoperative care

Postoperatively, the arm was placed in a Gilchrist’s sling for up to 6 weeks. Passive mobilization and pendulum exercises were allowed on day one postoperatively and drains were removed 2 days after surgery. Active Abduction and Flexion up to 30-40° was started 14 days after the operation. In the cases of K-wire fixation, the removal of material was planned about 6 weeks after the initial surgery. By this time the patient was allowed to do active flexion and abduction up to 70° resp. 90°. A higher range of motion was avoided due to the risk of material breakage in the KW-group. By the 7^th^ week after initial surgery, the range of active movement was extended. The patients were prohibited from performing activities that stressed the AC joint and working overhead until the 10^th^ week. Muscle strengthening exercises were delayed until the 12^th^ week.

### Statistics

The focus was on the timing of surgical treatment, duration and costs of hospital stay, diagnostic costs, operation time and operation costs (anesthesia, physician payment, material costs). Proceeds for stationary and outpatient treatment were recorded. Costs and proceeds were provided by the hospital’s Department for Financial Control. Statistics were carried out using SPSS (Version 11.5.1). The Kolmogorov-Smirnov-Test was used on all data to test for normal distribution. Metric data were compared using the Student t test. Descriptive results are demonstrated as the mean (range). The level of significance was defined as p = 0.05. Graphics were illustrated by using Windows EXCEL^®^.

## Results

All in all 80 patients with an AC-injury were treated during 2004 to 2011. In total, 41 patients met the inclusion criteria [TR: n = 18, m = 17, f = 1, mean age 37.3 (range 19-63 years); KW: n = 23, m = 21, f = 2, mean age 34.8 (range 17-56 years)]. The rest either suffered from polytrauma (n = 8), concomitant shoulder injury (n = 8) or received AC joint resection (n = 4). Further cost calculations could not be accomplished due to missing data (n = 19).

The TR group included 16 Rockwood III and 2 Rockwood type IV injuries. The KW group included 22 Rockwood III and 1 Rockwood IV lesions.

Most of the injuries were caused by sporting activities (n = 13), but were also caused by traffic accidents involving a car (n = 5), motorbike (n = 4) or bicycle (n = 8). Falls were another cause (n = 7). In 4 cases, the pathomechanism remained unclear.

Diagnostic, laboratory and radiology costs showed no significant differences.

**Figure 1:**
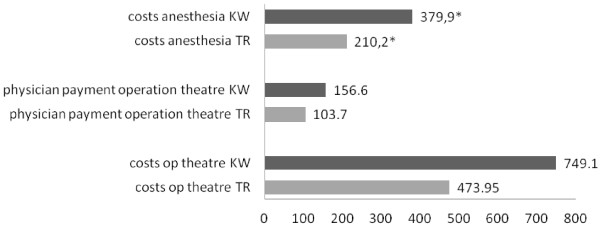
Costs of theater KW and Costs of theater TR in €, p = 0.016.

The period of time from injury to operation was similar in both groups (TR: 5.6 ±3.9d vs. KW: 7.3 ±4.3d, n.s). The operation time itself was also not significantly different, with a trend towards a shorter OR time in the TR group (TR: 64.3 ±19.8 vs. KW: 80.9 ±33.7 min, n.s.). A tendency for lower costs in operation theater use and physician payment was seen in the TR group, and the costs for anesthesia were significantly lower in the TR group (Figure 1).

Material costs were significantly higher in the TR group (340.0 ±123.7€) vs. the KW group (4€, p < 0.001). The remaining costs (from the accident and emergency department, physiotherapy, etc.) did not vary significantly between the two groups (TR: 74.61 ±31.1€ vs. KW: 90.3 ±7.9€, n.s.).

**Figure 2:**
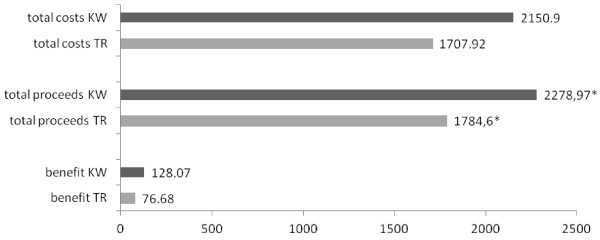
Costs, proceeds and benefit in €, p = 0.018.

Patients from the TR group left hospital earlier (TR: 2 ±1d vs. KW: 3.6 ±1.8d, p = 0.002) but produced higher costs during their stationary stay (TR: 906.5 ±67.6€ vs. KW: 856.2 ±12.9€, p = 0.044). Finally, total costs for treatment did not show any significant differences (TR: 1707.92 ±713.48€ vs. KW: 2150.9 ±75.41€, n.s.). Hospital proceeds were significantly higher in the KW group (TR: 1784.6 ±377.71€ vs. KW: 2279 ±411.22€, p = 0.021) but no significant financial benefit for either of the groups (TR 76.68 ±498.73€ vs. KD 128.07 ±372.55€, n.s.) was seen (Figure 2).

Although patients in the TR group completed their treatment earlier (49.4 ±30.7d vs 107.6 ±120.2d, n.s.) their appearance for follow-up visits was significantly higher (4.8 ±3.8 vs 1.5 ±2.4, p = 0.028). Proceeds for follow-up visits were higher in the TR group (TR: 99 ±32.7€ vs. KW: 21.1 ±0.5€, p = 0.019).

Severe complications (i.e. a fracture of the clavicle or nerve damage) occurred in neither of the groups. The early loss of reduction was seen in the TR group (n = 1) and migrating K-wires were recorded in 4 cases and led to the loss of reduction in one case. Impaired wound healing was seen in two cases within the TR group and impingement syndrome was recorded once within the KW group.

## Discussion

In 2004, a new pricing system for hospital services has been put in effect in Germany. So called Diagnosis-Related-Groups (DRG) should help to reduce the rising costs. Almost all the acute hospitals in Germany are obliged to use these fixed prices for their invoices regardless the type of insurance, foreign private or self-paying patients. For each of the DRG codes a specific economic case value has been prescribed, and this case value, multiplied with a base rate which is up to now specific for each hospital produces the price that may be billed for a specific patient. Nevertheless there has been a cost increase of about 21.7 billion euros in the general health care sector within the last 4 years, for which a number of reasons can be found. About 18 million patients approached hospital in 2010 and caused costs of 3,854 Euros per person, which was 2.2% more than in 2009 [20]. Employees (47.4 billion) and material costs (30.2 billion) were the main cost factors. Costs in both sectors increased (+3.4% and +3.3%) compared to the previous year. Educational costs (1 billion, +1.7%) and taxes (1.1 billion) therefore appear almost unimportant. Costs for non-stationary treatment were approximately 10.3 billion and included costs for treatment in the outpatients department, research costs and educational costs. Stationary costs were about 69.5 billion Euros [20].

The goal of this study was to evaluate the costs involved in the operative treatment of acromioclavicular luxation. It was found that costs resulting from the first medical contact in the accident and emergency department, as well as radiologic costs, did not vary between the two groups. Due to the same algorithm being used for all patients that presented with a typical appearance of ACJ-luxation, it was not expected that any major differences would be found in either the TR or the KW group during diagnosis. Once the indication for operative treatment was made, further diagnostic procedures were necessary. Analyzing costs for the laboratory did not reveal any significant differences. Only the diagnostic costs in cardiology varied minimally. Overall, patients that were included were very homogenous in age, gender, physical activity and injury pattern. Therefore, higher costs were not expected for any group, unlike patients with a physically-reduced status that would have the need for further pre-operative preparation.

It was found that patients from the TR group were operated on earlier. Most often patients that suffer from an acromioclavicular luxation will be able to be planned for surgery. While both techniques are available in our institution, a clear tendency towards the use of the tight rope technique was seen. This reconstruction technique represents a minimally-invasive method used to augment the torn conoid and trapezoid ligaments in acute ACJ separations [9]. Advantages are seen by the better cosmetic appearance as well as the minimized surgical trauma in comparison to open procedures. Furthermore, there is no need for implant removal as for techniques using the hook plate, K-wires or the Bosworth screw [6]. These facts might obviously help the patient in early decision-making for operative treatment. Therefore, the timing of the operation shifted towards the day of the injury.

A clear trend for shorter operation times was also seen in the TR group (TR: 64.3 ±19.8 min vs. KW: 80.9 ±33.7 min, n.s.). This finding was not significant, but patients that were treated by the tight rope device still had an incision to closure time that was about one fourth shorter than the patients treated with K-wires. This observation is supported by the significantly reduced costs for anesthesia in the TR group. The reasons for that can be found in the different techniques of both surgical methods. Two wires need to be placed to reduce rotation instability. Also, certain risks, such as the damage of neurovascular structures, must be minimized. Radiographic controls, cautious proceeding, tension banding and ligament suture is time-consuming. Due to the shorter operation time, pending surgical interventions can be processed earlier. In conclusion, more operations can be carried out through a limited period and an increase in financial turnover is possible. The positive value of optimizing the theater and bed capacities for operations was demonstrated [21,22].

The total physician payment (Anesthesiologist and Surgeon) did not show any significant differences per operation or per minute. Divergent findings in physician payment were not expected due to the fact that all physicians are employed by the hospital on the basis of regulated payment. These findings could vary from the costs and proceeds that emerge depending on the physicians’ employment status (i.e. self-employed) and insurance status of the patient (i.e. private insurance). Therefore, costs and proceeds may be interesting for insurances or hospitals letting operating theater capacities to external surgeons.

Material costs were significantly higher in the TR group (340.0 ±123.7€) vs. the KW group (4€, p < 0.001). K-wires do have a wide field of application, and surgeons from many operative fields have used them for years. Therefore, they are available separately or in small numbers and are placed via reusable drills. The tight rope repair kit contains non-recyclable materials that are developed for limited indications. Also single and double placed tight rope devices were not distinguished between. Out of the 18 patients operated on with the tight rope device, twelve were operated by the single tight rope technique (STR) while six received a double tight rope (DTR). Therefore, costs do vary at this point. A discussion regarding the two techniques is ongoing. Patzer et al. found that the DTR technique provided a lower CC distance compared to the STR technique [19]. These results were neither significant in difference of CC distance nor in scores [19]. In total, the collective group of patients described here presented a clear tendency for lower total operating theater costs in the TR group.

Patients from the TR group left hospital earlier (TR: 2 ±1d vs. KW: 3.6 ±1.8d, p = 0.002). The reasons for that could be the fact that the tight rope technique is a minimally-invasive procedure using small incisions, causing less soft tissue damage, being less invasive with regards to placing foreign material and offering a higher postoperative range of motion. Use of analgesia seems to be lower in the TR group. Early discharge after minimally-invasive surgery is common and also transferable to our patients [23,24]. However stationary costs were slightly higher in the TR group (TR: 906.5 ±67.6€ vs. KW: 856.2 ±12.9€, p = 0.044).

Costs are one point to be discussed. Another interesting aspect of cost analysis is the financial benefit of treating a specific disease or injury. While the complete costs were equal in this cohort (TR: 1707.92 ±713.48€ vs. KW: 2150.9 ±75.41€, n.s.), hospital proceeds were significantly higher in the KW group (TR: 1784.6 ±377.71€ vs. KW: 2279 ±411.22€, p = 0.021). This fact has to be discussed critically. Due to its novelty, the tight rope technique is not frequently used yet and therefore is not adequately reflected within the DRG system. Unlike the tight rope technique, K-wire fixation is more often performed by sewing the ruptured ligaments. Using either procedure will make a difference in coding the DRG. Also, patients from the TR group were discharged 1.5 times earlier, therefore not causing stationary costs that could be reimbursed. Nevertheless, analyzing costs and proceeds did not show a significant drawback by using either procedure (Figure 2). However, a strong point to keep in mind is the follow-up costs for K-wire removal once healing is achieved. A second surgical intervention is necessary in these patients and is either carried out in an ambulant or stationary situation. Even if costs and proceeds vary at this point, using K-wire fixation will cause follow-up costs, unlike using the tight rope technique.

Aftercare was identical in both groups. The costs for physiotherapy did not vary significantly, which can be tracked back to satisfying results on the one hand and limitations in prescribing follow-up treatment on the other. It was not expected to find huge variations in costs here. Due to the physical status of the patients and the good prognosis of the injury pattern, aftercare is also less complex than in other traumatic injuries.

Severe complications (i.e. a fracture of the clavicle or nerve damage) occurred in neither of the groups. The early loss of reduction was seen in the TR group (n = 1), but failures and loss of reduction are common problems when using the tight rope technique [25]. Ongoing studies are currently trying to identify the cause. One problem could be the indication, as it is possible that the suture is not adequate for hypermobile AC joint luxation [26]. Other publications have tried to reveal the outcome of using a double-bundle technique [18]. Migrating K-wires were recorded in 4 cases and led to the loss of reduction in one case. Impaired wound healing was seen in two cases within the TR group and impingement syndrome was recorded once within the KW group.

Overall, patients in the TR group tended to complete treatment earlier (49.4 ±30.7d vs 107.6 ±120.2d, n.s.), and their appearance for follow-up visits was increased (4.8 ±3.8 vs 1.5 ±2.4, p = 0.028). Proceeds for follow-up visits were higher in the TR group. The reason that patients in the KW group completed their treatment later can be explained by the second surgical intervention they received. K-wires need to be removed, which normally happens about six weeks after the first operation. Press et al. found that patients did not received surgery returned back to work after 0.8 months compared to 2.6 in those that were operated [27]. Our results show that treatment in the KW group lasted about 3.5 months. Treatment in the TR group was completed almost half of the time compared to the KW group and patients could return back to work. The higher rate of follow-up visits in the TR group is not reliable, due to the fact that patients were scheduled irregularly.

The limitations of this study are its retrospective design and the fact that, due to the DRG-based clearing system in Germany, it was not possible to clarify how higher stationary costs developed in the TR group. For the same reason, comparing overall proceeds using the TightRope™ device or K-wire fixation must be investigated carefully. Also the total number of 41 patients that were included is slightly low which mainly can be explained by the strict inclusion criteria of this study. According to our hypothesis we did focus on the costs that were caused while detailed clinical and radiological results fade into the background. Other study designs have to focalize on these endpoints.

## Conclusion

It was found that using the tight rope technique does offer advantages for the patient. The operation time tended to be shorter, an earlier discharge from hospital was possible and the treatment showed a trend to be completed faster. These are important findings from the patients’ point of view. The total operation theater costs also showed a favorable incline towards the tight rope technique. Also, an indirect influence on cost-containment can be assumed. However, from a financial point of view, using a tight rope device is not associated with excessive cost savings.

## Competing interests

The authors declare that there are no conflicts of interest including financial, consultant, institutional and other relationships. There’s no competing interest related to the company producing the TightRope™ device.

## Authors’ contributions

The work presented here involved the collaboration of all authors. KH, TD and MP defined the research theme and designed the methods, collected and analyzed the data, interpreted the results and wrote the paper. RMS and PK worked on interpretation and discussed the analyses, interpretation, and presentation. HCP gave critical and final approval. All authors have contributed to, seen and approved the manuscript.
